# Multiphase wavetrains, singular wave interactions and the emergence of the Korteweg–de Vries equation

**DOI:** 10.1098/rspa.2016.0456

**Published:** 2016-12

**Authors:** Daniel J. Ratliff, Thomas J. Bridges

**Affiliations:** Department of Mathematics, University of Surrey, Guildford GU2 7XH, UK

**Keywords:** nonlinear waves, wave action, modulation

## Abstract

Multiphase wavetrains are multiperiodic travelling waves with a set of distinct wavenumbers and distinct frequencies. In conservative systems, such families are associated with the conservation of wave action or other conservation law. At generic points (where the Jacobian of the wave action flux is non-degenerate), modulation of the wavetrain leads to the dispersionless multiphase conservation of wave action. The main result of this paper is that modulation of the multiphase wavetrain, when the Jacobian of the wave action flux vector is singular, morphs the vector-valued conservation law into the scalar Korteweg–de Vries (KdV) equation. The coefficients in the emergent KdV equation have a geometrical interpretation in terms of projection of the vector components of the conservation law. The theory herein is restricted to two phases to simplify presentation, with extensions to any finite dimension discussed in the concluding remarks. Two applications of the theory are presented: a coupled nonlinear Schrödinger equation and two-layer shallow-water hydrodynamics with a free surface. Both have two-phase solutions where criticality and the properties of the emergent KdV equation can be determined analytically.

## 1. Introduction

There is a multitude of physical examples where multiphase wavetrains arise naturally. In general, they are quasi-periodic solutions and so may have small divisor problems in establishing existence. However, in the case where there is some underlying symmetry, multiphase wavetrains can be characterized as relative equilibria and as such exist robustly in multiparameter families. For a vector-valued field, **u**(*x*,*t*), satisfying the Euler–Lagrange equation associated with a Lagrangian functional, an example of a multiphase wavetrain is a solution of the form

$$\text{u}(x,t)=\hat{\text{u}}(\theta_{1},\theta_{2}),\;\theta_{j}=k_{j}x+\omega_{j}t+\theta_{j}^{0},\;j=1,2,$$

where (*k*_1_,*k*_2_), (*ω*_1_,*ω*_2_) are the, in general distinct, wavenumbers and frequencies.

In this paper, we are interested in the modulation of multiphase wavetrains in conservative systems. This problem was first studied by Ablowitz & Benney [1], using Whitham modulation theory. They derived the conservation of wave action for scalar fields with two phases in detail, and showed how the theory generalized to *N*-phases. Examples in Ablowitz [2] show that, in general, one should expect small divisors, but weakly nonlinear solutions could still be obtained. However, for integrable systems, multiphase averaging and the Whitham equations are robust and rigorous, and a general theory can be obtained [3]. On the other hand, if the system is not integrable, but there is an *N*-fold symmetry, then again a theory for conservation of wave action can be developed without small divisors and smoothly varying *N*-phase wavetrains [4]. In essence, the conservation of wave action is replaced by the conservation law generated by the symmetry. There is now a vast literature on multiphase averaging and dispersionless Whitham theory, but we are not going to pursue this direction further as the interest here is in how dispersion can be generated in the modulation of multiphase wavetrains.

The strategy, in this paper, for generating dispersion in modulation equations deduced from the multiphase conservation of wave action is motivated by the theory for the single-phase case studied in [5–7], but has subtle new features. The starting point is a general class of partial differential equations (PDEs) generated by a Lagrangian

1.1 $$L(V)=\int_{t_{1}}^{t_{2}}\int_{x_{1}}^{x_{2}}L(V,V_{x},V_{t})\;dx\;dt,$$

where *V* (*x*,*t*) is a vector-valued function of (*x*,*t*) on the rectangle [*x*_1_,*x*_2_]×[*t*_1_,*t*_2_]. It is advantageous to first transform the Lagrangian density to multisymplectic form

1.2 $$L(Z)=\int_{t_{1}}^{t_{2}}\int_{x_{1}}^{x_{2}}\left[\frac{1}{2}\langle Z,\text{M}Z_{t}\rangle +\frac{1}{2}\langle Z,\text{J}Z_{x}\rangle -S(Z)\right]\;dx\;dt,$$

where now $Z\in R^{n}$ for each (*x*,*t*) and *n* is assumed to be even. The structure operators **M** and **J** are constant skew-symmetric *n*×*n* matrices and $S:R^{n}\to R$ is a given smooth function. The transformation from (1.1) to (1.2), which is effectively a double Legendre transform, is discussed in the previous papers [5–8]. The Euler–Lagrange equation deduced from the Lagrangian (1.2) takes the tidy form

1.3 $$\text{M}Z_{t}+\text{J}Z_{x}=\nabla S(Z),\;Z\in R^{n}.$$

Suppose there exists a two-phase solution

1.4 $$Z(x,t)=\hat{Z}(\theta ,\text{k},\omega ),\;\theta =\left(\begin{array}{c} \theta_{1}\\ \theta_{2} \end{array}\right),\;\text{k}=\left(\begin{array}{c} k_{1}\\ k_{2} \end{array}\right),\;\omega =\left(\begin{array}{c} \omega_{1}\\ \omega_{2} \end{array}\right),$$

satisfying (1.3) with

1.5 $$\theta_{j}=k_{j}x+\omega_{j}t+\theta_{j}^{0},\;j=1,2$$

and

$$\hat{Z}(\theta_{1}+2\pi ,\theta_{2},\cdot )=\hat{Z}(\theta_{1},\theta_{2},\cdot )=\hat{Z}(\theta_{1},\theta_{2}+2\pi ,\cdot ),\;\forall \;\text{k},\omega .$$

This two-phase solution can also be interpreted as an interaction between two waves. Associated with the two-phase solution is a vector-valued conservation law

1.6 $$A_{t}+B_{x}=0,\;A=\left(\begin{array}{c} A_{1}\\ A_{2} \end{array}\right),\;B=\left(\begin{array}{c} B_{1}\\ B_{2} \end{array}\right),$$

which may be conservation of wave action, or a conservation law associated, via Noether theory, to a two-dimensional Lie group symmetry of the PDE (1.3). Evaluation of the components of the conservation law (1.6) on the two-phase solutions generates mappings

1.7 (k,ω)↦A(k,ω)and(k,ω)↦B(k,ω).

The notation here is that (*A*,*B*) are the components of the wave action conservation law when considered as functions of (*x*,*t*) and (**A**,**B**) when they are considered as functions of (**k**,***ω***), with components

$$\text{A}(\text{k},\omega )=\left(\begin{array}{c} A_{1}(\text{k},\omega )\\ A_{2}(\text{k},\omega ) \end{array}\right)\;\text{and}\;\text{B}(\text{k},\omega )=\left(\begin{array}{c} B_{1}(\text{k},\omega )\\ B_{2}(\text{k},\omega ) \end{array}\right).$$

In the generic case, the mappings (1.7) are bijective. The interest in this paper is when the mapping **k**↦**B**(**k**,***ω***) fails to be bijective, in particular it is assumed that

1.8 $$\det[D_{\text{k}}\text{B}]=0,$$

at some **k**_0_(***ω***), and the zero eigenvalue is assumed to be simple with eigenvector $\zeta \in R^{2}$,

1.9 $$[D_{\text{k}}\text{B}]\zeta =0.$$

To simplify notation, the subscript on **k**_0_ is dropped, and it is clear in context whether **k**_0_ or **k** is intended. For each fixed ***ω***, the condition (1.8) defines a curve in **k** space which is called the criticality curve, or, as ***ω*** varies, the criticality surface. The matrix conditions (1.8)–(1.9) for emergence of dispersion generalize the scalar condition in the single-phase case. The condition (1.8) is a mathematical condition, but in §3f the connection with physical criticality is discussed.

Given the basic state the strategy is to modulate using an ansatz, motivated by the one-phase case in [5–7], but generalized to two phases

1.10 $$Z(x,t)=\hat{Z}(\theta +\varepsilon \phi ,\text{k}+\varepsilon^{2}\text{q},\omega +\varepsilon^{4}\Omega )+\varepsilon^{3}W(\theta ,X,T,\varepsilon ),$$

with slow time and space scales

1.11 $$X=\varepsilon x\;\text{and}\;T=\varepsilon^{3}t.$$

The ansatz (1.10) is substituted into (1.3), everything is expanded in Taylor series in *ε*, and solved order by order. The main result of this paper is that the *ε*^5^ equation is solvable if and only if the following equation is satisfied:

1.12 $$(D_{\text{k}}\text{A}+D_{\omega }\text{B})\zeta U_{T}+D_{\text{k}}^{2}\text{B}(\zeta ,\zeta )UU_{X}+\text{K}\;U_{XXX}+D_{\text{k}}\text{B}\;\alpha_{XX}=0,$$

where **K** and $\alpha \in R^{2}$ are 2-vectors and their definitions materialize in the theory. The scalar function *U* is obtained by projection

$$\text{Pq}:=U(X,T)\zeta ,\;\text{where}\;\text{P}:=∥\zeta {∥}^{-2}\zeta \zeta^{T}.$$

Equation (1.12) is quite remarkable. First, it is a vector Korteweg–de Vries (KdV) equation—almost. The extra fourth term changes it, and, as appears in the theory, the vector ***α*** is unknown at this stage. Second, the coefficients of the first, second and fourth terms are all expressed purely in terms of derivatives of the components of the conservation law and the eigenvector ***ζ***, with the latter determined by (1.9).

On the other hand, D_**k**_**B** is singular by assumption (1.8) and symmetric, so if the inner product of ***ζ*** with equation (1.12) is taken, the last term drops out, and a scalar KdV equation emerges. Projection of (1.12) onto the complement of the kernel of D_**k**_**B** provides an equation determining ***α***, which would be used at the next order.

Although this ansatz (1.10) looks like a straightforward generalization of the ansatz in the single-phase case, there are subtle non-trivial differences in the multiphase theory. The Jordan chain theory here (developed in §3b) intertwines the theory in the single phase case with the new eigenvector *ζ* in (1.9). It is not *a priori* clear how two solvability conditions generate one equation, and here it is the projection and the essential role of ***α*** that are important (there is no analogue in the single-phase theory).

In summary, the theory starts with a conservative PDE, generated by a Lagrangian in canonical form (1.1)–(1.2), with a four-parameter family of two-phase wavetrains (1.4). When this four-parameter family has a simple degeneracy (1.8), the modulation ansatz (1.10) satisfies the Euler–Lagrange equation (1.3) up to fifth order in *ε* when (*U*,***α***) satisfy (1.12). Projection of (1.12) in the direction of the kernel of D_**k**_**B** then generates a scalar KdV equation

1.13 $$\zeta^{T}(D_{\text{k}}\text{A}+D_{\omega }\text{B})\zeta U_{T}+\zeta^{T}D_{\text{k}}^{2}\text{B}(\zeta ,\zeta )UU_{X}+\zeta^{T}\text{K}U_{XXX}=0.$$

In other words, it is the simple degeneracy (1.8) which is responsible for generating dispersion in the modulation of multiphase wavetrains, and the coefficients of the emergent KdV equation can be described in terms of the geometry of the mappings (1.7).

There are many examples of conservative PDEs with multiphase wavetrains. In this paper, we include two representative examples. The first is two-layer shallow-water hydrodynamics with a free surface, and the second example is a coupled nonlinear Schrödinger (NLS) equation. In both cases, there is a natural two-parameter symmetry group, the first is associated with conservation of mass in each layer, and the coupled NLS has a natural toral symmetry. A multiphase wavetrain can be explicitly constructed in both cases and parameter values identified where the determinant condition (1.8) is satisfied. Potential generalizations are discussed in the concluding remarks section.

## 2. Governing equations and wavetrain properties

The governing equation is (1.3) with **M**^*T*^=−**M**, **J**^*T*^=−**J**, and, for simplicity, **J** is assumed to be invertible. Non-invertible **J** just requires an additional assumption on the kernel of **J**.

Suppose there exists a two-phase solution of the form (1.4). Substitution into (1.3) gives the following governing equation for the two-phase wavetrain:

2.1 $$(\omega_{1}\text{M}+k_{1}\text{J}){\hat{Z}}_{\theta_{1}}+(\omega_{2}\text{M}+k_{2}\text{J}){\hat{Z}}_{\theta_{2}}=\nabla S(\hat{Z}).$$

In the absence of symmetry, solution of this problem may encounter small divisors. However, we assume throughout that $\hat{Z}$ is a smooth function of ***θ***, **k** and ***ω***. A class of systems for which this is true in general is symmetric systems when the multiphase wavetrains are relative equilibria associated with the symmetry, and both the examples in this paper have this property.

Linearizing (1.3) about the two-phase solution leads to the linear operator

2.2 $$\text{L}\text{v}=D^{2}S(\hat{Z})\text{v}-(\omega_{1}\text{M}+k_{1}\text{J}){\text{v}}_{\theta_{1}}-(\omega_{2}\text{M}+k_{2}\text{J}){\text{v}}_{\theta_{2}}.$$

One can verify that this operator is formally self-adjoint with respect to the inner product

2.3 $$\langle \langle \cdot ,\cdot \rangle \rangle ={\left(\frac{1}{2\pi }\right)}^{2}\int_{0}^{2\pi }\int_{0}^{2\pi }\langle \cdot ,\cdot \rangle \;d\theta_{1}\;d\theta_{2},$$

where 〈⋅,⋅〉 is the standard inner product on $R^{n}$.

In the modulation analysis, equations for the derivatives of $\hat{Z}$, with respect to the phases, the wavenumbers and the frequencies, are needed, and we find that

$$\begin{array}{rl} & \theta_{i}:\;(\omega_{1}\text{M}+k_{1}\text{J}){\hat{Z}}_{\theta_{1}\theta_{i}}+(\omega_{2}\text{M}+k_{2}\text{J}){\hat{Z}}_{\theta_{i}\theta_{2}}=D^{2}S(\hat{Z}){\hat{Z}}_{\theta_{i}},\\ & k_{i}:\;(\omega_{1}\text{M}+k_{1}\text{J}){\hat{Z}}_{\theta_{1}k_{i}}+\text{J}{\hat{Z}}_{\theta_{i}}+(\omega_{2}\text{M}+k_{2}\text{J}){\hat{Z}}_{\theta_{1}k_{i}}=D^{2}S(\hat{Z}){\hat{Z}}_{k_{i}},\\ & \omega_{i}:\;(\omega_{1}\text{M}+k_{1}\text{J}){\hat{Z}}_{\theta_{1}\omega_{i}}+\text{M}{\hat{Z}}_{\theta_{i}}+(\omega_{2}\text{M}+k_{2}\text{J}){\hat{Z}}_{\theta_{1}\omega_{i}}=D^{2}S(\hat{Z}){\hat{Z}}_{\omega_{i}}. \end{array}$$

These can be summarized as

2.4*a* $$\begin{array}{rl} \text{L}{\hat{Z}}_{\theta_{i}} & =0, \end{array}$$

2.4*b* $$\begin{array}{rl} \text{L}{\hat{Z}}_{k_{i}} & =\text{J}{\hat{Z}}_{\theta_{i}} \end{array}$$

2.4*c* $$\begin{array}{rl} \;\mathrm{and}\;\text{L}{\hat{Z}}_{\omega_{i}} & =\text{M}{\hat{Z}}_{\theta_{i}},\;i=1,2. \end{array}$$

The first of these equations highlights that the zero eigenvalue of **L** is not simple, and so we make the assumption that

2.5 $$\ker(\text{L})=\text{span}\{{\hat{Z}}_{\theta_{1}},{\hat{Z}}_{\theta_{2}}\}$$

and it is no larger. The second and third equations ((2.4*b*) and (2.4*c*)) suggest that there are two different Jordan chains, and these two chains will feature prominently in the modulation analysis. We also require the second derivatives with respect to wavenumber, and they can be summarized as

2.6*a* $$\text{L}{\hat{Z}}_{k_{i}k_{i}}=2\text{J}{\hat{Z}}_{\theta_{i}k_{i}}-D^{3}S(\hat{Z})({\hat{Z}}_{k_{i}},{\hat{Z}}_{k_{i}}),\;i=1,2$$

and

2.6*b* $$\text{L}{\hat{Z}}_{k_{1}k_{2}}=\text{J}{\hat{Z}}_{\theta_{2}k_{1}}+\text{J}{\hat{Z}}_{\theta_{1}k_{2}}-D^{3}S(\hat{Z})({\hat{Z}}_{k_{1}},{\hat{Z}}_{k_{2}}).$$

The assumption (2.5) along with the formal self-adjointness of **L** give the solvability condition for

2.7 $$\text{L}W=F\;\text{as}\;\langle \langle {\hat{Z}}_{\theta_{1}},F\rangle \rangle =\langle \langle {\hat{Z}}_{\theta_{2}},F\rangle \rangle =0.$$

### (a) Conservation laws

When the Lagrangian is in canonical form (1.2), the components of the conservation law evaluated on the two-phase wavetrain, whether the conservation laws are deduced from symmetry or they are components of wave action conservation, have a geometrical form and can be readily deduced. The canonical Lagrangian (1.2), evaluated on the two-phase wave train and averaged, is

$$L(\text{k},\omega )=\frac{1}{{\left(2\pi \right)}^{2}}\int_{0}^{2\pi }\int_{0}^{2\pi }\left[\sum_{j=1}^{2}\left[\frac{1}{2}\omega_{j}\langle \hat{Z},\text{M}{\hat{Z}}_{\theta_{j}}\rangle +\frac{1}{2}k_{j}\langle \hat{Z},\text{J}{\hat{Z}}_{\theta_{j}}\rangle \right]-S(\hat{Z})\right]d\theta_{1}\;d\theta_{2}.$$

The wave action vector, or symmetry-induced density, is

$$\text{A}(\text{k},\omega )=\left(\begin{array}{c} A_{1}\\ A_{2} \end{array}\right):=\left(\begin{array}{c} L_{\omega_{1}}\\ L_{\omega_{2}} \end{array}\right)=\frac{1}{2}\left(\begin{array}{c} \left\langle \langle \text{M}{\hat{Z}}_{\theta_{1}},\hat{Z}\rangle \right\rangle \\ \left\langle \langle \text{M}{\hat{Z}}_{\theta_{2}},\hat{Z}\rangle \right\rangle \end{array}\right),$$

and the associated flux vector is

$$\text{B}(\text{k},\omega )=\left(\begin{array}{c} B_{1}\\ B_{2} \end{array}\right):=\left(\begin{array}{c} L_{k_{1}}\\ L_{k_{2}} \end{array}\right)=\frac{1}{2}\left(\begin{array}{c} \left\langle \langle \text{J}{\hat{Z}}_{\theta_{1}},\hat{Z}\rangle \right\rangle \\ \left\langle \langle \text{J}{\hat{Z}}_{\theta_{2}},\hat{Z}\rangle \right\rangle \end{array}\right).$$

By definition,

$$\begin{array}{rl} D_{\text{k}}\text{A} & =\left(\begin{array}{cc} \partial_{k_{1}}A_{1} & \partial_{k_{2}}A_{1}\\ \partial_{k_{1}}A_{2} & \partial_{k_{2}}A_{2} \end{array}\right)=D_{\omega }{\text{B}}^{T},\;D_{\text{k}}\text{B}=\left(\begin{array}{cc} \partial_{k_{1}}B_{1} & \partial_{k_{2}}B_{1}\\ \partial_{k_{1}}B_{2} & \partial_{k_{2}}B_{2} \end{array}\right),\\ D_{\text{k}}^{2}\text{B} & =\left(\begin{array}{cc} \partial_{k_{1}k_{1}}B_{1} & \partial_{k_{2}k_{1}}B_{1}\\ \partial_{k_{1}k_{1}}B_{2} & \partial_{k_{2}k_{1}}B_{2} \end{array}\;|\;\begin{array}{cc} \partial_{k_{1}k_{2}}B_{1} & \partial_{k_{2}k_{2}}B_{1}\\ \partial_{k_{1}k_{2}}B_{2} & \partial_{k_{2}k_{2}}B_{2} \end{array}\right). \end{array}$$

The entries of these tensors are related to solutions via

2.8*a* $$\begin{array}{rl} \partial_{k_{j}}A_{i} & =\langle \langle \text{M}{\hat{Z}}_{\theta_{i}},{\hat{Z}}_{k_{j}}\rangle \rangle , \end{array}$$

2.8*b* $$\begin{array}{rl} \partial_{k_{j}}B_{i} & =\langle \langle \text{J}{\hat{Z}}_{\theta_{i}},{\hat{Z}}_{k_{j}}\rangle \rangle \end{array}$$

2.8*c* $$\begin{array}{rl} \;\mathrm{and}\;\partial_{k_{j}k_{m}}B_{i} & =\langle \langle \text{J}{\hat{Z}}_{\theta_{i}k_{m}},{\hat{Z}}_{k_{j}}\rangle \rangle +\langle \langle \text{J}{\hat{Z}}_{\theta_{i}},{\hat{Z}}_{k_{j}k_{m}}\rangle \rangle . \end{array}$$

Note that

2.9 $$\partial_{k_{i}}B_{j}=\langle \langle \text{J}{\hat{Z}}_{\theta_{j}},{\hat{Z}}_{k_{i}}\rangle \rangle =\langle \langle \text{L}{\hat{Z}}_{k_{j}},{\hat{Z}}_{k_{i}}\rangle \rangle =\langle \langle {\hat{Z}}_{k_{j}},\text{L}{\hat{Z}}_{k_{i}}\rangle \rangle =\langle \langle {\hat{Z}}_{k_{j}},\text{J}{\hat{Z}}_{\theta_{i}}\rangle \rangle =\partial_{k_{j}}B_{i}$$

as well as

$$\partial_{k_{j}}A_{i}=\langle \langle \text{M}{\hat{Z}}_{\theta_{i}},{\hat{Z}}_{k_{i}}\rangle \rangle =\langle \langle {\hat{Z}}_{\omega_{i}},\text{J}{\hat{Z}}_{k_{j}}\rangle \rangle =\partial_{\omega_{i}}B_{j}.$$

We say that a conservation law is critical in the multiphase case if the zero determinant condition (1.8) is satisfied. The details of the modulation analysis when (1.8) is satisfied now follow.

## 3. Multiphase modulation leading to the Korteweg–de Vries equation

With the assumption (1.8), the proposed modulation ansatz is (1.10), with

3.1 $$\text{q}=\left(\begin{array}{c} q_{1}\\ q_{2} \end{array}\right):={\left(\begin{array}{c} \phi_{1}\\ \phi_{2} \end{array}\right)}_{X}\;\text{and}\;\Omega =\left(\begin{array}{c} \Omega_{1}\\ \Omega_{2} \end{array}\right):={\left(\begin{array}{c} \phi_{1}\\ \phi_{2} \end{array}\right)}_{T}.$$

These arise from the phase consistency conditions relating *k*_*i*_,*ω*_*i*_ to *θ*_*i*_. In particular, the above definitions impose that **q**_*T*_=***Ω***_*X*_, which is vector-valued conservation of waves. The strategy is to substitute the ansatz into the Euler–Lagrange equation (1.3), expand everything in Taylor series in *ε* and solve the equations at each order in *ε*. The Taylor expansion of *W* is

$$\varepsilon^{3}W=\varepsilon^{3}W_{3}+\varepsilon^{4}W_{4}+\varepsilon^{5}W_{5}+\cdots .$$

The full details of the Taylor expansions are lengthy and cumbersome, and are a generalization of [5–7], and so only a summary of the key steps of the expansion are recorded below, with particular attention on the essential differences owing to the multiphase wavetrain.

The *ε*^0^ equation just returns the equation for the basic state (2.1). The *ε*^1^ equation is

$$\phi_{1}\text{L}{\hat{Z}}_{\theta_{1}}+\phi_{2}\text{L}{\hat{Z}}_{\theta_{2}}=0.$$

Both the linear operator terms present in the above are zero by (2.4*a*), and so this is trivially satisfied, for any *ϕ*_1_ and *ϕ*_2_, by the basic state. The *ε*^2^ terms are

$$q_{1}\text{L}{\hat{Z}}_{k_{1}}+q_{2}\text{L}{\hat{Z}}_{k_{2}}-{\left(\phi_{1}\right)}_{X}\text{J}{\hat{Z}}_{\theta_{1}}-{\left(\phi_{2}\right)}_{X}\text{J}{\hat{Z}}_{\theta_{2}}=0.$$

Using (2.4*b*), we have

$$(q_{1}-{\left(\phi_{1}\right)}_{X})\text{J}{\hat{Z}}_{\theta_{1}}+(q_{2}-{\left(\phi_{2}\right)}_{X})\text{J}{\hat{Z}}_{\theta_{2}}=0.$$

Because $\text{J}{\hat{Z}}_{\theta_{1}}\neq 0$ and $\text{J}{\hat{Z}}_{\theta_{2}}\neq 0$ (or else the multiphase wavetrain would degenerate to a single-phase wavetrain or an equilibrium), we recover that *q*_1_=(*ϕ*_1_)_*X*_ and *q*_2_=(*ϕ*_2_)_*X*_.

### (a) Third order

At third order, the equations become more interesting. We find that

3.2 $$\text{L}W_{3}={\left(q_{1}\right)}_{X}\text{J}{\hat{Z}}_{k_{1}}+{\left(q_{2}\right)}_{X}\text{J}{\hat{Z}}_{k_{2}}.$$

Apply the solvability condition (2.7),

$$\begin{array}{rl} {\left(q_{1}\right)}_{X}\langle \langle {\hat{Z}}_{\theta_{1}},\text{J}{\hat{Z}}_{k_{1}}\rangle \rangle +{\left(q_{2}\right)}_{X}\langle \langle {\hat{Z}}_{\theta_{1}},\text{J}{\hat{Z}}_{k_{2}}\rangle \rangle & =0\\ {\left(q_{1}\right)}_{X}\langle \langle {\hat{Z}}_{\theta_{2}},\text{J}{\hat{Z}}_{k_{1}}\rangle \rangle +{\left(q_{2}\right)}_{X}\langle \langle {\hat{Z}}_{\theta_{2}},\text{J}{\hat{Z}}_{k_{2}}\rangle \rangle & =0, \end{array}$$

or equivalently, after using (2.4*b*),

3.3 $$\text{0}=\left(\begin{array}{cc} \partial_{k_{1}}B_{1} & \partial_{k_{2}}B_{1}\\ \partial_{k_{1}}B_{2} & \partial_{k_{2}}B_{2} \end{array}\right)\left(\begin{array}{c} {\left(q_{1}\right)}_{X}\\ {\left(q_{2}\right)}_{X} \end{array}\right)=[D_{\text{k}}\text{B}]{\text{q}}_{X}.$$

To avoid triviality of the modulation (i.e. when (*q*_1_)_*X*_=(*q*_2_)_*X*_=0), the requirement to proceed to the next order is that the 2×2 matrix D_**k**_**B** must be singular. In the case where det[D_**k**_**B**]≠0, and so **q**_*X*_=0 leads to *W*_3_=0 (mod the kernel of D_**k**_**B**), the theory fails and dispersion does not emerge. When det[D_**k**_**B**]≠0, the relevant modulation equation is dispersionless multiphase Whitham equations, albeit with a time scale *T*=*εt*.

It is at this point in the analysis where the assumption (1.8) becomes important. With the assumption that the zero eigenvalue of D_**k**_**B** is simple with eigenvector (1.9), it follows from (3.3) that we must have

3.4 $${\text{q}}_{X}=\left(\begin{array}{c} {\left(q_{1}\right)}_{X}\\ {\left(q_{2}\right)}_{X} \end{array}\right)=U_{X}\zeta ,$$

for some new slowly varying function *U*(*X*,*T*). In principle, *U*(*X*,*T*) also depends on *ε*, but here only the leading-order term is needed so *U*(*X*,*T*)≡*U*(*X*,*T*,*ε*)|_*ε*=0_. Integrating (3.4),

3.5 q(X,T,⋅)=U(X,T)ζ+V(T).

The function of integration *V* (*T*) can be neglected as it does not affect the leading-order KdV equation that arises at fifth order.

The equation for *W*_3_ in (3.2) can now be cast into the form

3.6 $$\text{L}W_{3}=U_{X}(\zeta_{1}\text{J}{\hat{Z}}_{k_{1}}+\zeta_{2}\text{J}{\hat{Z}}_{k_{2}}),$$

with solution

3.7 $$W_{3}=\alpha_{1}(X,T){\hat{Z}}_{\theta_{1}}+\alpha_{2}(X,T){\hat{Z}}_{\theta_{2}}+U_{X}\xi_{5},$$

where ***α***=(*α*_1_,*α*_2_) is unknown at this point, and *ξ*_5_ is the solution of

3.8 $$\text{L}\xi_{5}=\zeta_{1}\text{J}{\hat{Z}}_{k_{1}}+\zeta_{2}\text{J}{\hat{Z}}_{k_{2}}.$$

Equation (3.8) is solvable due to the fact that ***ζ***∈Ker[D_**k**_**B**]. The solution for *ξ*_5_ coalesces the two Jordan chains in (2.4*c*), leading to a 2⊕4 Jordan block structure. We digress here to identify further properties of the symplectic Jordan chain arising in this case.

### (b) Jordan chain theory interlude—multiple blocks

Jordan chain theory plays a key role in the modulation theory for the emergence of the KdV equation for the one-phase case in [5–7]. Here, a non-trivial generalization of that theory is required. There are two Jordan chains, but they intertwine. With the assumption (2.5), the zero eigenvalue of **L** has geometric multiplicity two. Hence, there are two Jordan blocks

$$\begin{array}{rl} \text{L}{\hat{Z}}_{\theta_{1}} & =0\\ \text{L}{\hat{Z}}_{k_{1}} & =\text{J}{\hat{Z}}_{\theta_{1}} \end{array}\}\;\text{and}\;\left\{ \begin{array}{rl} \;\text{L}{\hat{Z}}_{\theta_{2}} & =0\\ \;\text{L}{\hat{Z}}_{k_{2}} & =\text{J}{\hat{Z}}_{\theta_{2}}. \end{array}\right.$$

However, the existence of a solution to (3.8) suggests that a longer Jordan chain will exist. Introduce the labelling

$$\xi_{1}={\hat{Z}}_{\theta_{1}},\;\xi_{2}={\hat{Z}}_{\theta_{2}},\;\xi_{3}={\hat{Z}}_{k_{1}},\;\xi_{4}={\hat{Z}}_{k_{2}}.$$

The existence of a solution to (3.8) defines *ξ*_5_ and, because all symplectic Jordan chains have an even number of elements, the next element is *ξ*_6_ satisfying

3.9 $$\text{L}\xi_{6}=\text{J}\xi_{5}.$$

By changing basis, the 2⊕4 structure of the two Jordan chains emerges

$$\begin{array}{rl} \text{L}(-\zeta_{2}\xi_{1}+\zeta_{1}\xi_{2}) & =0\\ \text{L}(-\zeta_{2}\xi_{3}+\zeta_{1}\xi_{4}) & =\text{J}(-\zeta_{2}\xi_{1}+\zeta_{1}\xi_{2}) \end{array}\}\;\text{and}\;\left\{ \begin{array}{rl} \text{L}(\zeta_{1}\xi_{1}+\zeta_{2}\xi_{2}) & =0\\ \text{L}(\zeta_{1}\xi_{3}+\zeta_{2}\xi_{4}) & =\text{J}(\zeta_{1}\xi_{1}+\zeta_{2}\xi_{2})\\ \text{L}\xi_{5} & =\text{J}(\zeta_{1}\xi_{3}+\zeta_{2}\xi_{4})\\ \text{L}\xi_{6} & =\text{J}\xi_{5}. \end{array}\right.$$

The left-hand chain terminates at two, due to the fact that zero is a simple eigenvalue of D_**k**_**B**. It is assumed that the right-hand chain terminates at four. Define

3.10 $$K_{j}:=\langle \langle \text{J}\xi_{j},\xi_{6}\rangle \rangle ,\;j=1,2.$$

Then, non-solvability of **L***ξ*_7_=**J***ξ*_6_ ensures that $K_{1}^{2}+K_{2}^{2}>0$.

### (c) Fourth order

After simplification, absorbing the relevant terms into the linear operator, and replacing **q** with *U****ζ***, we find that

3.11 $$\text{L}{\tilde{W}}_{4}+\Omega_{1}\text{L}{\hat{Z}}_{\omega_{1}}-{\left(\phi_{1}\right)}_{T}\text{M}{\hat{Z}}_{\theta_{1}}+\Omega_{2}\text{L}{\hat{Z}}_{\omega_{2}}-{\left(\phi_{2}\right)}_{T}\text{M}{\hat{Z}}_{\theta_{2}}-U_{XX}\text{J}\xi_{5}=0,$$

with

3.12 $$\begin{array}{rl} {\tilde{W}}_{4} & =W_{4}-{\left(\alpha_{1}\right)}_{X}{\hat{Z}}_{k_{1}}-{\left(\alpha_{2}\right)}_{X}{\hat{Z}}_{k_{2}}-\zeta_{1}U\alpha_{1}{\hat{Z}}_{\theta_{1}\theta_{1}}-(\zeta_{2}U\alpha_{1}+\zeta_{1}U\alpha_{2}){\hat{Z}}_{\theta_{1}\theta_{2}}\\ & \;-\zeta_{2}U\alpha_{2}{\hat{Z}}_{\theta_{2}\theta_{2}}-\phi_{1}U_{X}{\left(\xi_{5}\right)}_{\theta_{1}}-\phi_{2}U_{X}{\left(\xi_{5}\right)}_{\theta_{2}}. \end{array}$$

The time derivatives in *ϕ*_1_,*ϕ*_2_ cancel the *Ω*_1_,*Ω*_2_ terms by (2.4*c*). Hence, using the top of the Jordan chain,

3.13 $${\tilde{W}}_{4}=U_{XX}\xi_{6}+\gamma_{1}(X,T)\xi_{1}+\gamma_{1}(X,T)\xi_{2}.$$

Therefore, the complete solution at this order for *W*_4_ is

3.14 $$\begin{array}{rl} W_{4} & =\gamma_{1}\xi_{1}+\gamma_{2}\xi_{2}+{\left(\alpha_{1}\right)}_{X}\xi_{3}+{\left(\alpha_{2}\right)}_{X}\xi_{4}\\ & \;+\zeta_{1}U\alpha_{1}{\hat{Z}}_{\theta_{1}\theta_{1}}+(\zeta_{2}U\alpha_{1}+\zeta_{1}U\alpha_{2}){\hat{Z}}_{\theta_{1}\theta_{2}}+\zeta_{2}U\alpha_{2}{\hat{Z}}_{\theta_{2}\theta_{2}}\\ & \;+\phi_{1}U_{X}{\left(\xi_{5}\right)}_{\theta_{1}}+\phi_{2}U_{X}{\left(\xi_{5}\right)}_{\theta_{2}}+U_{XX}\xi_{6}, \end{array}$$

where *γ*_1_(*X*,*T*) and *γ*_2_(*X*,*T*) are arbitrary at this stage.

### (d) Fifth order

After simplification, the *ε*^5^ terms are

3.15 $$\begin{array}{rl} \text{L}{\tilde{W}}_{5} & =\zeta_{1}U_{T}(\text{M}{\hat{Z}}_{k_{1}}+\text{J}{\hat{Z}}_{\omega_{1}})+\zeta_{2}U_{T}(\text{M}{\hat{Z}}_{k_{2}}+\text{J}{\hat{Z}}_{\omega_{2}})+U_{XXX}\text{J}\xi_{6}\\ & \;-\zeta_{2}UU_{X}(D^{3}S(\hat{Z})({\hat{Z}}_{k_{2}},\xi_{5})-\text{J}{\left(\xi_{5}\right)}_{\theta_{2}}-\zeta_{2}\text{J}{\hat{Z}}_{k_{2}k_{2}}-\zeta_{1}\text{J}{\hat{Z}}_{k_{1}k_{2}})\\ & \;-\zeta_{1}UU_{X}(D^{3}S(\hat{Z})({\hat{Z}}_{k_{1}},\xi_{5})-\text{J}{\left(\xi_{5}\right)}_{\theta_{1}}-\zeta_{2}\text{J}{\hat{Z}}_{k_{1}k_{2}}-\zeta_{1}\text{J}{\hat{Z}}_{k_{1}k_{1}})\\ & \;+{\left(\alpha_{1}\right)}_{XX}\text{J}{\hat{Z}}_{k_{1}}+{\left(\alpha_{2}\right)}_{XX}\text{J}{\hat{Z}}_{k_{2}}, \end{array}$$

where ${\tilde{W}}_{5}$ includes all terms that can be absorbed into **L** as in (3.12). However, the explicit expression for ${\tilde{W}}_{5}$ is not needed as the solvability condition delivers the required vector KdV equation (1.12).

The key terms on the right-hand side are proportional to *U*_*T*_, *U*_*XXX*_, *UU*_*X*_ and ***α***_*XX*_, so the form of (1.12) is emerging. Application of the two conditions for solvability (2.7) produces two equations. The solvability conditions are applied term by term. The coefficients of the *U*_*T*_ term involve

$$\langle \langle {\hat{Z}}_{\theta_{i}},\text{M}{\hat{Z}}_{k_{j}}+\text{J}{\hat{Z}}_{\omega_{j}}\rangle \rangle =-\langle \langle \text{M}{\hat{Z}}_{\theta_{i}},{\hat{Z}}_{k_{j}}\rangle \rangle -\langle \langle \text{J}{\hat{Z}}_{\theta_{i}},{\hat{Z}}_{\omega_{j}}\rangle \rangle =-\partial_{k_{j}}A_{i}-\partial_{\omega_{j}}B_{i}.$$

Therefore, defining the vector **e** by

$$\text{e}:=-\left(\begin{array}{c} \left\langle \langle {\hat{Z}}_{\theta_{1}},\zeta_{1}(\text{M}{\hat{Z}}_{k_{1}}+\text{J}{\hat{Z}}_{\omega_{1}})+\zeta_{2}(\text{M}{\hat{Z}}_{k_{2}}+\text{J}{\hat{Z}}_{\omega_{2}})\rangle \right\rangle \\ \left\langle \langle {\hat{Z}}_{\theta_{2}},\zeta_{1}(\text{M}{\hat{Z}}_{k_{1}}+\text{J}{\hat{Z}}_{\omega_{1}})+\zeta_{2}(\text{M}{\hat{Z}}_{k_{2}}+\text{J}{\hat{Z}}_{\omega_{2}})\rangle \right\rangle \end{array}\right),$$

the vector coefficient of *U*_*T*_ is

$$\begin{array}{rl} \text{e} & =\left(\begin{array}{c} \zeta_{1}(\partial_{k_{1}}A_{1}+\partial_{\omega_{1}}B_{1})+\zeta_{2}(\partial_{k_{2}}A_{1}+\partial_{\omega_{2}}B_{1})\\ \zeta_{1}(\partial_{k_{1}}A_{1}+\partial_{\omega_{1}}B_{2})+\zeta_{2}(\partial_{k_{2}}A_{2}+\partial_{\omega_{2}}B_{2}) \end{array}\right)\\ & =\left(\begin{array}{cc} \left(\partial_{k_{1}}A_{1}+\partial_{\omega_{1}}B_{1}\right) & \left(\partial_{k_{2}}A_{1}+\partial_{\omega_{2}}B_{1}\right)\\ \left(\partial_{k_{1}}A_{1}+\partial_{\omega_{1}}B_{2}\right) & \left(\partial_{k_{2}}A_{2}+\partial_{\omega_{2}}B_{2}\right) \end{array}\right)\left(\begin{array}{c} \zeta_{1}\\ \zeta_{2} \end{array}\right)\\ & =[D_{\text{k}}\text{A}+D_{\omega }\text{B}]\zeta . \end{array}$$

For the coefficient of *U*_*XXX*_, use (3.10) and define $\text{K}={\left(K_{1},K_{2}\right)}^{T}$. Solvability of the ***α***_*XX*_ terms is similar to the construction in §3*a* and solvability gives a term [D_**k**_**B**]***α***_*XX*_. By combining terms, and multiplying by −1, solvability of (3.15) gives

3.16 $$\text{e}\;U_{T}+\text{f}\;UU_{X}+\text{K}\;U_{XXX}+[D_{\text{k}}\text{B}]\alpha_{XX}=0.$$

To confirm that this is the same as (1.12), it remains to show that

3.17 $$\text{f}=D^{2}\text{B}(\zeta ,\zeta ):={\frac{d^{2}}{ds^{2}}\text{B}(\text{k}+s\zeta ,\omega )|}_{s=0}.$$

With **f**=( *f*_1_,*f*_2_), solvability of (3.15) gives, for *j*=1,2, that

3.18 $$\begin{array}{rl} -f_{j} & =-\zeta_{2}\langle \langle {\hat{Z}}_{\theta_{j}},(D^{3}S(\hat{Z})({\hat{Z}}_{k_{2}},\xi_{5})-\text{J}{\left(\xi_{5}\right)}_{\theta_{2}}-\zeta_{2}\text{J}{\hat{Z}}_{k_{2}k_{2}}-\zeta_{1}\text{J}{\hat{Z}}_{k_{1}k_{2}})\rangle \rangle \\ & \;-\zeta_{1}\langle \langle {\hat{Z}}_{\theta_{j}},(D^{3}S(\hat{Z})({\hat{Z}}_{k_{1}},\xi_{5})-\text{J}{\left(\xi_{5}\right)}_{\theta_{1}}-\zeta_{2}\text{J}{\hat{Z}}_{k_{1}k_{2}}-\zeta_{1}\text{J}{\hat{Z}}_{k_{1}k_{1}})\rangle \rangle . \end{array}$$

To relate these terms to derivatives of **B**(**k**,***ω***), use (2.8*c*) to obtain

$$\begin{array}{rl} \zeta_{1}\partial_{k_{1}k_{1}}B_{j}+\zeta_{2}\partial_{k_{1}k_{2}}B_{j} & =\zeta_{1}(\langle \langle \text{J}{\hat{Z}}_{\theta_{j}k_{1}},{\hat{Z}}_{k_{1}}\rangle \rangle +\langle \langle \text{J}{\hat{Z}}_{\theta_{j}},{\hat{Z}}_{k_{1}k_{1}}\rangle \rangle )+\zeta_{2}(\langle \langle \text{J}{\hat{Z}}_{\theta_{j}k_{2}},{\hat{Z}}_{k_{1}}\rangle \rangle +\langle \langle \text{J}{\hat{Z}}_{\theta_{j}},{\hat{Z}}_{k_{1}k_{2}}\rangle \rangle )\\ & =-\langle \langle {\hat{Z}}_{\theta_{j}k_{1}},\zeta_{1}\text{J}{\hat{Z}}_{k_{1}}+\zeta_{2}\text{J}{\hat{Z}}_{k_{2}}\rangle \rangle -\langle \langle {\hat{Z}}_{\theta_{j}},\zeta_{1}\text{J}{\hat{Z}}_{k_{1}k_{1}}+\zeta_{2}\text{J}{\hat{Z}}_{k_{1}k_{2}}\rangle \rangle \\ & =-\langle \langle {\hat{Z}}_{\theta_{j}k_{1}},\text{L}\xi_{5}\rangle \rangle -\langle \langle {\hat{Z}}_{\theta_{j}},\zeta_{1}\text{J}{\hat{Z}}_{k_{1}k_{1}}+\zeta_{2}\text{J}{\hat{Z}}_{k_{1}k_{2}}\rangle \rangle . \end{array}$$

Differentiating (2.4*a*) with respect to *k*_*j*_, $\text{L}{\hat{Z}}_{\theta_{i}k_{j}}=-D^{3}S(\hat{Z})({\hat{Z}}_{k_{j}},{\hat{Z}}_{\theta_{i}})+\text{J}{\hat{Z}}_{\theta_{i}\theta_{j}}$, hence

$$\begin{array}{rl} \zeta_{1}\partial_{k_{1}k_{1}}B_{j}+\zeta_{2}\partial_{k_{1}k_{2}}B_{j} & =-\langle \langle \text{L}{\hat{Z}}_{\theta_{j}k_{1}},\xi_{5}\rangle \rangle -\langle \langle {\hat{Z}}_{\theta_{j}},\zeta_{1}\text{J}{\hat{Z}}_{k_{1}k_{1}}+\zeta_{2}\text{J}{\hat{Z}}_{k_{1}k_{2}}\rangle \rangle \\ & =\langle \langle D^{3}S(\hat{Z})({\hat{Z}}_{k_{1}},{\hat{Z}}_{\theta_{j}})-\text{J}{\hat{Z}}_{\theta_{j}\theta_{1}},\xi_{5}\rangle \rangle -\langle \langle {\hat{Z}}_{\theta_{j}},\zeta_{1}\text{J}{\hat{Z}}_{k_{1}k_{1}}+\zeta_{2}\text{J}{\hat{Z}}_{k_{1}k_{2}}\rangle \rangle \\ & =\langle \langle {\hat{Z}}_{\theta_{j}},D^{3}S(\hat{Z})({\hat{Z}}_{k_{1}},\xi_{5})-\text{J}{\left(\xi_{5}\right)}_{\theta_{1}}-\zeta_{1}\text{J}{\hat{Z}}_{k_{1}k_{1}}-\zeta_{2}\text{J}{\hat{Z}}_{k_{1}k_{2}}\rangle \rangle . \end{array}$$

Similarly, it can be shown that

$$\zeta_{1}\partial_{k_{1}k_{2}}B_{j}+\zeta_{2}\partial_{k_{2}k_{2}}B_{j}=\langle \langle {\hat{Z}}_{\theta_{j}},D^{3}S(\hat{Z})({\hat{Z}}_{k_{2}},\xi_{5})-\zeta_{1}\text{J}{\hat{Z}}_{k_{1}k_{2}}-\zeta_{2}\text{J}{\hat{Z}}_{k_{2}k_{2}}-\text{J}{\left(\xi_{5}\right)}_{\theta_{2}}\rangle \rangle .$$

Substitute these expressions into (3.18)

$$f_{j}=\zeta_{1}(\zeta_{1}\partial_{k_{1}k_{1}}B_{j}+\zeta_{2}\partial_{k_{1}k_{2}}B_{j})+\zeta_{2}(\zeta_{1}\partial_{k_{1}k_{2}}B_{j}+\zeta_{2}\partial_{k_{2}k_{2}}B_{j}),\;j=1,2,$$

and so

$$\text{f}=\left(\begin{array}{c} \zeta_{1}^{2}\partial_{k_{1}k_{1}}B_{1}+2\zeta_{1}\zeta_{2}\partial_{k_{1}k_{2}}B_{1}+\zeta_{2}^{2}\partial_{k_{2}k_{2}}B_{1}\\ \zeta_{1}^{2}\partial_{k_{1}k_{1}}B_{2}+2\zeta_{2}\zeta_{2}\partial_{k_{1}k_{2}}B_{j}+\zeta_{2}^{2}\partial_{k_{2}k_{2}}B_{2} \end{array}\right)=D^{2}\text{B}(\zeta ,\zeta ).$$

Projection of (3.16) in the direction of the kernel of D_**k**_**B** then gives the scalar KdV equation (1.13).

In the one-phase case, the modulation equation arising at fifth order is precisely the KdV equation. Here, the intermediate equation (3.16) arises and it is indeterminate, because ***α***_*XX*_ is unknown at this order. Hence, it is not a coupled KdV equation. It is at best an inhomogeneous coupled KdV equation with an indeterminate inhomogeneity. It is here that the eigenvector ***ζ*** associated with the zero eigenvalue of D_**k**_**B** plays a central role. Projection of (3.16) in the direction of ***ζ*** then delivers a fully determined scalar KdV equation. Projection of (3.16) in the direction of the complement of the kernel of D_**k**_**B** gives a defining equation for ***α***_*XX*_, which is not needed until the next order.

The above analysis also gives an indication of how a true fully determined coupled KdV equation can arise: when D_**k**_**B** has a double-zero eigenvalue with two independent eigenvectors, with the system once again emerging when one projects in the direction of the kernel.

### (e) Modulation of steady multiphase wavetrains

The reduction to KdV is essentially the same when the multiphase wavetrain is steady

3.19 $$Z(x,t)=\hat{Z}(\theta_{1},\theta_{2},\text{k}),\;\text{k}=\left(\begin{array}{c} k_{1}\\ k_{2} \end{array}\right),$$

with $\theta_{j}=k_{j}x+\theta_{j}^{0},\;j=1,2$. In this case, there is no frequency modulation, but the resulting equation is the same as (1.12) with the first coefficient replaced by

$$D_{\text{k}}\text{A}+D_{\omega }\text{B}=D_{\text{k}}\text{A}+D_{\text{k}}{\text{A}}^{T}.$$

Then, all coefficients depend only on **k** and so can be computed from the steady solution. This simplification is useful in examples.

The justification of this claim is as follows. The (*ϕ*_*i*_)_*T*_ terms in (3.11) now are no longer cancelled with the *Ω*_*i*_ terms, as these are not present, and so the additional solvability requirement is

3.20 $$\langle \langle {\hat{Z}}_{\theta_{i}},\text{M}{\hat{Z}}_{\theta_{j}}\rangle \rangle =0,\;i,j=1,2.$$

This has to be true, because the zero eigenvalue is of even multiplicity and this is part of the Jordan chain formed by **M** instead of **J**. Therefore, there exists *κ*_*i*_ such that

$$\text{L}\kappa_{i}=\text{M}{\hat{Z}}_{\theta_{i}}.$$

This means that an additional term of the form ${\left(\phi_{i}\right)}_{T}\text{M}{\hat{Z}}_{\theta_{i}}$ appears as part of the solution in (3.14). The presence of these terms at the next order shows the vector coefficient **e** is now given by

$$\begin{array}{rl} \text{e} & =-\left(\begin{array}{c} \left\langle \langle {\hat{Z}}_{\theta_{1}},\zeta_{1}(\text{M}{\hat{Z}}_{k_{1}}+\text{J}\kappa_{1})+\zeta_{2}(\text{M}{\hat{Z}}_{k_{2}}+\text{J}\kappa_{2})\rangle \right\rangle \\ \left\langle \langle {\hat{Z}}_{\theta_{2}},\zeta_{1}(\text{M}{\hat{Z}}_{k_{1}}+\text{J}\kappa_{1})+\zeta_{2}(\text{M}{\hat{Z}}_{k_{2}}+\text{J}\kappa_{2})\rangle \right\rangle \end{array}\right)\\ & =D_{\text{k}}\text{A}\zeta +\left(\begin{array}{c} \left\langle \langle \text{J}{\hat{Z}}_{\theta_{1}},\zeta_{1}\kappa_{1}+\zeta_{2}\kappa_{2}\rangle \right\rangle \\ \left\langle \langle \text{J}{\hat{Z}}_{\theta_{2}},\zeta_{1}\kappa_{1}+\zeta_{2}\kappa_{2}\rangle \right\rangle \end{array}\right)\\ & =D_{\text{k}}\text{A}\zeta +\left(\begin{array}{c} \left\langle \langle {\hat{Z}}_{k_{1}},\zeta_{1}\text{M}{\hat{Z}}_{\theta_{1}}+\zeta_{2}\text{M}{\hat{Z}}_{\theta_{2}}\rangle \right\rangle \\ \left\langle \langle {\hat{Z}}_{k_{2}},\zeta_{1}\text{M}{\hat{Z}}_{\theta_{1}}+\zeta_{2}\text{M}{\hat{Z}}_{\theta_{2}}\rangle \right\rangle \end{array}\right)\\ & =D_{\text{k}}\text{A}\zeta +D_{\text{k}}\text{A}\zeta^{T} \end{array}$$

as required.

### (f) From mathematical criticality to physical criticality

The emergence of the KdV equation owing to degeneracy of the Jacobian D_**k**_**B** is a mathematical result. The result is based on the abstract properties of a Lagrangian, and is deduced without any recourse to physical input. One interpretation of the importance of the mapping **k**→**B**(**k**,***ω***) can be seen by characterizing the Euler–Lagrange equation (2.1) as a constrained variational principle: multiphase wavetrains are critical points of the Lagrangian taking the wave action and wave action flux as constraints, then frequency and wavenumber are Lagrange multipliers. The values of **k**,***ω*** in the resulting solution $\hat{Z}(\theta ,\text{k},\omega )$ of the Euler–Lagrange equation (2.1) are determined by the values of the constraints. Therefore, the mappings (**k**,***ω***)↦(**A**,**B**) need to be non-degenerate in order to solve for (**k**,***ω***) in terms of the values of the constraint sets. Breakdown of this mapping, det[D_**k**_**B**]=0, we call (mathematical) criticality, although we generally use the term ‘criticality’ without qualification.

All the steps in the theory follow from mathematical considerations. Hence, it is all the more remarkable that it is an excellent model for capturing criticality in physical systems. The connection is easy to see in simple models such as one-layer shallow-water flow where criticality corresponds to Froude number unity. The Froude number unity condition can than be re-characterized as the appearance of a zero eigenvalue which, in turn, can be related to mathematical criticality (see §8 of [5]). In the fluid mechanics literature, criticality, in more complicated systems, is often associated with the appearance of zero eigenvalues (see [9] for an extensive discussion of this viewpoint). It is the appearance of zero eigenvalues that connects the two theories: zero eigenvalues are an essential part of both mathematical and physical criticality.

While the connection can be made fairly precise in fluid mechanics, in other systems, such as the coupled NLS equations arising as a model for Bose–Einstein condensates and nonlinear optics, the connection is not as clear because of the absence of a concept of physical criticality. Nevertheless, the mathematical theory applies to give results which then have physical implications. Below, two examples are given. The first from fluid mechanics shows how the theory of this paper gives a new mechanism and simplified derivation of the emergence of KdV in two-layer shallow-water hydrodynamics. The second example shows how a single KdV equation can emerge at criticality for coupled NLS equations, showing how the mathematical theory can evoke new results for the physical system, without the need to understand the physical mechanism behind the criticality condition for NLS.

## 4. Example 1: two-layer shallow-water flow

A natural application of the multiphase theory presented here is the case of shallow-water hydrodynamics with two layers of differing density bounded above by a free surface. The conservation laws in this case are mass conservation in each layer, and they are associated with the potential symmetry. The basic multiphase wavetrain in this case is just uniform flow (constant depth and velocity in each layer).

The governing equations for this system are

4.1*a* $$\begin{array}{rl} & {\left(\rho_{1}\eta \right)}_{t}+{\left(\rho_{1}\eta u_{1}\right)}_{x}=0, \end{array}$$

4.1*b* $$\begin{array}{rl} & {\left(\rho_{2}\chi \right)}_{t}+{\left(\rho_{2}\chi u_{2}\right)}_{x}=0, \end{array}$$

4.1*c* $$\begin{array}{rl} & {\left(\rho_{1}u_{1}\right)}_{t}+{\left(\frac{\rho_{1}}{2}u_{1}^{2}+g\rho_{1}\eta +g\rho_{2}\chi \right)}_{x}=a_{11}\eta_{xxx}+a_{12}\chi_{xxx} \end{array}$$

4.1*d* $$\begin{array}{rl} \;\mathrm{and}\; & {\left(\rho_{2}u_{2}\right)}_{t}+{\left(\frac{\rho_{2}}{2}u_{2}^{2}+g\rho_{2}\eta +g\rho_{2}\chi \right)}_{x}=a_{21}\eta_{xxx}+a_{22}\chi_{xxx}, \end{array}$$

with

$$\begin{array}{rl} a_{11} & =-\frac{1}{3}\rho_{1}g\eta_{0}^{2}-\rho_{2}g\eta_{0}\chi_{0}-\frac{1}{2}g\chi_{0}^{2},\\ a_{12} & =a_{21}=-\frac{1}{6}\rho_{2}g\eta_{0}^{2}-\frac{1}{4}\rho_{2}g\eta_{0}\chi_{0}-\frac{\rho_{2}^{2}}{2\rho_{1}}g\eta_{0}\chi_{0}-\frac{5}{12}\rho_{2}g\chi_{0}^{2},\\ a_{22} & =-\frac{\rho_{2}^{2}}{2\rho_{1}}g\eta_{0}\chi_{0}-\frac{1}{3}\rho_{2}g\chi_{0}^{2}. \end{array}$$

In these equations, *ρ*_1_, *η*, *u*_1_ are the density, depth and horizontal velocity in the lower layer, and *ρ*_2_, *χ*, *u*_2_ are the density, depth, and horizontal velocity in the upper layer. In the dispersion coefficients, *η*_0_ and *χ*_0_ are quiescent depths in the two layers. The dispersionless version of these equations is derived in Baines [10], and the dispersive terms are derived in Donaldson [11] (see also [12]).

The first two equations (4.1*a*) and (4.1*b*) are the system’s conservation laws and the symmetry associated with them is a constant shift of the velocity potentials which are defined by *u*_1_=(*ψ*_1_)_*x*_ and *u*_2_=(*ψ*_2_)_*x*_. Steady multiphase wavetrains associated with this symmetry take the form

$$\psi_{1}=\theta_{1}:=k_{1}x+\theta_{1}^{0}\;\text{and}\;\psi_{2}=\theta_{2}:=k_{2}x+\theta_{2}^{0},$$

where *k*_1_ and *k*_2_ are constant velocities in each layer. Substitution into the governing equations requires that *k*_1_ and *k*_2_ satisfy

4.2 $$\frac{1}{2}\rho_{1}k_{1}^{2}+\rho_{1}g\eta_{0}+\rho_{2}g\chi_{0}=R_{1}\;\text{and}\;\frac{1}{2}\rho_{2}k_{2}^{2}+\rho_{2}g\eta_{0}+\rho_{2}g\chi_{0}=R_{2},$$

where *R*_1_,*R*_2_ are constants of integration (Bernoulli constants in each layer). These two equations are used to express *η*_0_ and *χ*_0_ in terms of *k*_1_ and *k*_2_ in the conservation laws.

### (a) Conservation laws and criticality

The first two equations of this system (4.1*a*) and (4.1*b*) form the conservation laws for the system. Therefore, we have

4.3 $$A_{1}=\rho_{1}\eta ,\;A_{2}=\rho_{2}\chi ,\;B_{1}=\rho_{1}\eta u_{1},\;B_{2}=\rho_{2}\chi u_{2}.$$

Evaluating them on the basic state, and differentiating, gives the following necessary condition for the emergence of the KdV equation:

$$\det[D_{k}\text{B}]=\left|(\begin{array}{cc} \rho_{1}\eta_{0}-\frac{\rho_{1}k_{1}^{2}}{g(1-r)} & \frac{\rho_{2}k_{1}k_{2}}{g(1-r)}\\ \frac{\rho_{2}k_{1}k_{2}}{g(1-r)} & \rho_{2}\chi_{0}-\frac{\rho_{2}k_{2}^{2}}{g(1-r)} \end{array})\right|=0,$$

where *r*=*ρ*_2_/*ρ*_1_, which upon expansion is

4.4 $$\rho_{1}\rho_{2}\eta_{0}\chi_{0}(1-r-F_{1}^{2})(1-r-F_{2}^{2})-\frac{\rho_{2}^{2}k_{1}^{2}k_{2}^{2}}{g^{2}}=0\;\Rightarrow \;(1-F_{1}^{2})(1-F_{2}^{2})=r,$$

where we have introduced the Froude numbers

4.5 $$F_{1}^{2}=\frac{k_{1}^{2}}{g\eta_{0}},\;F_{2}^{2}=\frac{k_{2}^{2}}{g\chi_{0}}.$$

The condition for the emergence of KdV agrees with the classical criticality condition found in the literature [13–15]. The sign of det[D_**k**_**B**] in [14] was linked to the stability, with det[D_**k**_**B**]<0 being necessary (but not sufficient) for flow instability. This loss of stability was shown to lead to the presence of hydraulic jumps within the flow. Assume that the trace of D_**k**_**B** is non-zero, and so the zero eigenvalue is simple with eigenvector

$$\zeta =\left(\begin{array}{c} -\rho_{2}k_{1}k_{2}\\ g\rho_{1}\eta_{0}(1-r-F_{1}^{2}) \end{array}\right).$$

### (b) Emergence of Korteweg–de Vries at criticality

The relevant coefficient matrices for the vector KdV equation (1.12) are

$$\begin{array}{rl} D_{\text{k}}\text{A} & =\frac{1}{g(1-r)}\left(\begin{array}{cc} -\rho_{1}k_{1} & \rho_{2}k_{2}\\ \rho_{2}k_{1} & -\rho_{2}k_{2} \end{array}\right),\\ D_{\text{k}}^{2}\text{B} & =\frac{1}{2g(1-r)}\left(\begin{array}{cc} -6\rho_{1}k_{1} & 2\rho_{2}k_{2}\\ 2\rho_{2}k_{2} & 2\rho_{2}k_{1} \end{array}\;|\;\begin{array}{cc} 2\rho_{2}k_{2} & 2\rho_{2}k_{1}\\ 2\rho_{2}k_{1} & -6\rho_{2}k_{2} \end{array}\right). \end{array}$$

Now, we project to obtain the coefficients of the reduced KdV equation, using the theory of §3. The first term projects to

$$\zeta^{T}(D_{\text{k}}\text{A}+D_{\text{k}}{\text{A}}^{T})\zeta =-2g^{2}\rho_{1}^{2}\rho_{2}\chi_{0}\eta_{0}^{2}(1-r-F_{1}^{2})\left(\frac{k_{1}}{g\eta_{0}}(1-F_{2}^{2})+\frac{k_{2}}{g\chi_{0}}(1-F_{1}^{2})\right).$$

For the second derivative

$$\zeta^{T}D_{\text{k}}^{2}\text{B}(\zeta ,\zeta )=3\;g^{2}\rho_{1}^{3}\rho_{2}k_{2}\eta_{0}^{2}{\left(1-r\right)}^{2}(1-r-F_{1}^{2})(\chi_{0}r(1-F_{2}^{2})F_{1}^{2}-\eta_{0}{\left(1-F_{1}^{2}\right)}^{2}F_{2}^{2}).$$

Because the dispersion vector **K** is a coefficient of a linear term, it can be calculated using the dispersion relation (associated with the linearization about the basic state) or using the Jordan chain. The details are omitted and we just state the result,

$$\text{K}=-\frac{1}{g(1-r)}\left(\begin{array}{c} \rho_{1}k_{1}T_{1}\\ \rho_{2}k_{2}T_{2} \end{array}\right),$$

with

$$\begin{array}{rl} T_{1} & =\frac{\zeta_{2}(a_{11}r+(1+r)a_{12}+a_{22})k_{2}-\zeta_{1}(a_{11}+2a_{12}+a_{22})k_{1}}{g\rho_{1}(1-r)},\\ T_{2} & =\frac{\zeta_{1}(a_{11}r+(1+r)a_{12}+a_{22})k_{1}-\zeta_{2}(a_{11}r^{2}+2a_{12}r+a_{22})k_{2}}{g\rho_{2}(1-r)}. \end{array}$$

This projects to

$$\zeta_{1}K_{1}+\zeta_{2}K_{2}=g\rho_{1}^{2}\eta_{0}^{2}\chi_{0}(1-r-F_{1}^{2})(a_{11}r(1-F_{2}^{2})-2ra_{12}+(1-F_{1}^{2})a_{22}).$$

Therefore, the reduced scalar KdV equation after simplification is

4.6 $$\begin{array}{rl} & \rho_{2}\chi_{0}\left(\frac{k_{1}}{g\eta_{0}}(1-F_{2}^{2})+\frac{k_{2}}{g\chi_{0}}(1-F_{1}^{2})\right)U_{T}\\ & \;-\frac{3}{2}\rho_{1}\rho_{2}k_{2}(\chi_{0}r(1-F_{2}^{2})F_{1}^{2}-\eta_{0}{\left(1-F_{1}^{2}\right)}^{2}F_{2}^{2})UU_{X}\\ & \;-\frac{\chi_{0}}{2g}(a_{11}r(1-F_{2}^{2})-2ra_{12}+(1-F_{1}^{2})a_{22})U_{XXX}=0. \end{array}$$

The purpose here is to show that, once the singularity condition (1.8) is established, the coefficients in the emergent KdV equation are determined via elementary calculations. The derivation of a single KdV for two-layer flow is not new (see [12,15] and references therein; the coefficient of the nonlinearity here agrees with that in [12,15]). It is how the KdV arises that is new and of interest here. Further details, including physical implications, the multisymplectic formulation of the problem and discussion of validity will be given elsewhere. The multisymplectic formulation can be obtained as the tensor product of the multisymplectic formulation of the single-layer shallow-water equations in §8 of [5].

## 5. Example 2: NLS × NLS → KdV reduction

Another example where a two-phase wavetrain arises naturally is the coupled NLS equation. To illustrate, we use a coupled NLS equation which appears in the theory of water waves [16] and in models for Bose–Einstein condensates [17]

5.1*a* $$i\partial_{t}\Psi_{1}+\alpha_{1}{\left(\Psi_{1}\right)}_{xx}+(\beta_{11}|\Psi_{1}{|}^{2}+\beta_{12}|\Psi_{2}{|}^{2})\Psi_{1}=0$$

and

5.1*b* $$i\partial_{t}\Psi_{2}+\alpha_{2}{\left(\Psi_{2}\right)}_{xx}+(\beta_{21}|\Psi_{1}{|}^{2}+\beta_{22}|\Psi_{2}{|}^{2})\Psi_{2}=0,$$

for complex-valued functions *Ψ*_1_(*x*,*t*) and *Ψ*_2_(*x*,*t*) and real constants *α*_*i*_, *β*_*ij*_, with *β*_21_=*β*_12_. For convenience, define *β*=*β*_11_*β*_22_−*β*_12_*β*_21_≠0. There is a natural toral symmetry in that (*e*^*iθ*_1_^*Ψ*_1_,*e*^*iθ*_2_^*Ψ*_2_) is a solution for any (*θ*_1_,*θ*_2_)∈*S*^1^×*S*^1^ whenever (*Ψ*_1_,*Ψ*_2_) is a solution. A two-phase wavetrain associated with this symmetry is

5.2 $$\Psi_{1}(x,t)=\Psi_{1}^{\left(0\right)}e^{i\theta_{1}},\;\Psi_{2}(x,t)=\Psi_{1}^{\left(0\right)}e^{i\theta_{2}},$$

where the phases are taken to be steady: $\theta_{1}=k_{1}x+\theta_{1}^{0}$ and $\theta_{2}=k_{2}x+\theta_{2}^{0}$. Substitution of (5.2) into (5.1) requires **k** and the amplitudes to be related by

$$|\Psi_{1}^{\left(0\right)}{|}^{2}=\frac{1}{\beta }(\beta_{22}\alpha_{1}k_{1}^{2}-\beta_{12}\alpha_{2}k_{2}^{2}),\;|\Psi_{2}^{\left(0\right)}{|}^{2}=\frac{1}{\beta }(\beta_{11}\alpha_{2}k_{2}^{2}-\beta_{21}\alpha_{1}k_{1}^{2}).$$

In this section, the multiphase modulation theory of the paper is used to show that there is a large range of parameters where the coupled NLS equation can be reduced to a single KdV equation. As far as we are aware, this is the first time that NLS×NLS→KdV has been obtained. On the other hand, there are results in the literature reducing NLS×NLS→KdV×KdV owing to Brazhnyi & Konotop [18]. However, reduction to a coupled KdV requires additional parameters, and so will be more rare than the reduction to a single KdV here (see comments at the end of §3d).

### (a) Conservation laws and criticality

The components of the conservation law associated with the toral symmetry are

$$A_{1}=\frac{1}{2}|\Psi_{1}{|}^{2},\;A_{2}=\frac{1}{2}|\Psi_{2}{|}^{2},\;B_{1}=\alpha_{1}ℑ(\Psi_{1}^{*}{\left(\Psi_{1}\right)}_{x})\;\mathrm{and}\;B_{2}=\alpha_{2}ℑ(\Psi_{1}^{*}{\left(\Psi_{2}\right)}_{x}),$$

where an asterisk denotes complex conjugation and ℑ denotes the imaginary part of the bracketed expression. Evaluate (*A*,*B*) on the basic state

$$\text{A}(\text{k})=\frac{1}{2}\left(\begin{array}{c} \frac{1}{\beta }(\beta_{22}\alpha_{1}k_{1}^{2}-\beta_{12}\alpha_{2}k_{2}^{2})\\ \frac{1}{\beta }(\beta_{11}\alpha_{2}k_{2}^{2}-\beta_{21}\alpha_{1}k_{1}^{2}) \end{array}\right)\;\text{and}\;\text{B}(\text{k})=\left(\begin{array}{c} \frac{\alpha_{1}k_{1}}{\beta }(\beta_{22}\alpha_{1}k_{1}^{2}-\beta_{12}\alpha_{2}k_{2}^{2})\\ \frac{\alpha_{2}k_{2}}{\beta }(\beta_{11}\alpha_{2}k_{2}^{2}-\beta_{21}\alpha_{1}k_{1}^{2}) \end{array}\right).$$

Differentiating **B**(**k**)

$$D_{\text{k}}\text{B}=\left(\begin{array}{cc} \alpha_{1}|\Psi_{1}^{\left(0\right)}{|}^{2}(1+\beta_{22}E_{1}^{2}) & -\frac{2\alpha_{1}\alpha_{2}k_{1}k_{2}\beta_{12}}{\beta }\\ -\frac{2\alpha_{1}\alpha_{2}k_{1}k_{2}\beta_{12}}{\beta } & \alpha_{2}|\Psi_{2}^{\left(0\right)}{|}^{2}(1+\beta_{11}E_{2}^{2}) \end{array}\right),$$

where

5.3 $$E_{1}^{2}=\frac{2\alpha_{1}k_{1}^{2}}{\beta |\Psi_{1}^{\left(0\right)}{|}^{2}},\;E_{2}^{2}=\frac{2\alpha_{2}k_{2}^{2}}{\beta |\Psi_{2}^{\left(0\right)}{|}^{2}}.$$

The zero determinant condition (1.8) then requires

5.4 $$(1+\beta_{22}E_{1}^{2})(1+\beta_{11}E_{2}^{2})-\beta_{12}\beta_{21}E_{1}^{2}E_{2}^{2}=0.$$

Suppose the trace of D_**k**_**B** is non-zero and choose the eigenvector as

5.5 $$\zeta =\left(\begin{array}{c} \frac{2\alpha_{1}\alpha_{2}k_{1}k_{2}\beta_{12}}{\beta }\\ \alpha_{1}|\Psi_{1}^{\left(0\right)}{|}^{2}(1+\beta_{22}E_{1}^{2}) \end{array}\right).$$

The condition det[D_**k**_**B**]<0 has been shown to relate to the stability of these wavetrains. Examples of this instability criterion have been derived for specific systems by Roskes [16] and Law *et al.* [19]. A general result for conservative systems in terms of the conservation of wave action was derived in [4]. The condition det[D_**k**_**B**]=0 is therefore of interest both as a necessary condition for emergence of the KdV equation and as an instability boundary.

### (b) Emergence of Korteweg–de Vries at criticality

The relevant matrices needed for the emergent vector KdV equation (1.12) are

5.6 $$\begin{array}{rl} D_{\text{k}}\text{A} & =\frac{1}{\beta }\left(\begin{array}{cc} \alpha_{1}\beta_{22}k_{1} & -\alpha_{2}\beta_{12}k_{2}\\ -\alpha_{1}\beta_{12}k_{1} & \alpha_{2}\beta_{11}k_{2} \end{array}\right)\\ \;\mathrm{and}\;D_{\text{k}}^{2}\text{B} & =\frac{2}{\beta }\left(\begin{array}{cc} 3\alpha_{1}^{2}\beta_{22}k_{1} & -\alpha_{1}\alpha_{2}\beta_{12}k_{2}\\ -\alpha_{1}\alpha_{2}\beta_{12}k_{2} & -\alpha_{1}\alpha_{2}\beta_{12}k_{1} \end{array}\;|\;\begin{array}{cc} -\alpha_{1}\alpha_{2}\beta_{12}k_{2} & -\alpha_{1}\alpha_{2}\beta_{12}k_{1}\\ -\alpha_{1}\alpha_{2}\beta_{12}k_{1} & 3\alpha_{2}^{2}\beta_{11}k_{2} \end{array}\right). \end{array}\}$$

The first of these projects to

5.7 $$\zeta^{T}(D_{\text{k}}\text{A}+D_{\text{k}}{\text{A}}^{T})\zeta =\alpha_{3}(|\Psi_{2}^{\left(0\right)}{|}^{2}(\beta_{22}+\beta E_{2}^{2})k_{1}+|\Psi_{1}^{\left(0\right)}{|}^{2}(\beta_{11}+\beta E_{1}^{2})k_{2}),$$

where $\alpha_{3}=2\alpha_{1}^{2}\alpha_{2}\beta^{-1}|\Psi_{1}^{\left(0\right)}{|}^{2}(1+\beta_{22}E_{1}^{2})$. The second matrix has the projection

5.8 $$\zeta^{T}D_{\text{k}}^{2}\text{B}(\zeta ,\zeta )=3\alpha_{1}\alpha_{2}\alpha_{3}|\Psi_{1}^{\left(0\right)}{|}^{2}(|\Psi_{1}^{\left(0\right)}{|}^{2}(1+\beta_{22}E_{1}^{2})(\beta_{11}+\beta E_{1}^{2})-|\Psi_{2}^{\left(0\right)}{|}^{2}(1+\beta_{11}E_{2}^{2})\beta_{12}).$$

The dispersion coefficient ***ζ***^*T*^**K** can be calculated, using the dispersion relation from the linearization or by constructing explicitly the Jordan chain. The details are lengthy and will be given elsewhere. The result is

5.9 $$\begin{array}{rl} \zeta^{T}\text{K} & =\frac{1}{4}\alpha_{3}(\beta_{11}\beta_{22}E_{2}^{2}E_{1}^{2}(\alpha_{1}\beta_{22}|\Psi_{2}^{\left(0\right)}{|}^{2}+\alpha_{2}\beta_{11}|\Psi_{1}^{\left(0\right)}{|}^{2})+\alpha_{2}E_{1}^{2}\beta_{12}^{2}|\Psi_{1}^{\left(0\right)}{|}^{2}\\ & \;\;+E_{1}^{2}(\alpha_{1}\beta_{22}^{2}|\Psi_{2}^{\left(0\right)}{|}^{2}+\alpha_{2}\beta_{12}^{2}|\Psi_{1}^{\left(0\right)}{|}^{2})+E_{2}^{2}(\alpha_{2}\beta_{11}^{2}|\Psi_{1}^{\left(0\right)}{|}^{2}+\alpha_{1}\beta_{12}^{2}|\Psi_{2}^{\left(0\right)}{|}^{2})). \end{array}$$

Substitution of (5.7)–(5.9) into (1.13) then delivers the emergent scalar KdV equation in this case. Further details, including physical implications, the multisymplectic formulation of the problem and discussion of validity are given elsewhere. The multisymplectic formulation can be obtained as the tensor product of the multisymplectic formulation of the single NLS equation in §9 of [5].

## 6. Concluding remarks

This paper has shown that systems generated by a Lagrangian that have two conservation laws and possess two-phase wavetrain solutions can lead to the emergence of a scalar KdV equation, when the mapping from wavenumber space into the flux vector is singular. It appears that the theory will generalize to *m*-dimensional Lie groups with *m*>1 when the group is Abelian, with the condition that D_**k**_**B** have a simple zero eigenvalue, regardless of the dimension *m*. If the zero eigenvalue is not simple, then potentially a true vector-valued KdV equation is expected to emerge, but emergence of the coupled KdV is higher codimension, that is, it requires additional parameters.

Analysis of systems with a one-parameter Lie group has shown that, with additional conditions, other equations are generated by the modulation, e.g. the two-way Boussinesq equation [7] and the KP equation [12] in the 2+1 case. That theory should also generalize to the multiphase case. In the case of the Boussinesq equation, we would expect this to occur when the time term in the scalar KdV equation vanishes, which implies that the system

$$D_{\text{k}}\text{B}\alpha_{XX}=-(D_{\text{k}}\text{A}+D_{\omega }\text{B})\zeta$$

is solvable. With this additional condition and a suitable rescaling, one expects a similar approach to that here will lead to a scalar two-way Boussinesq.

The analysis presented here appears to generalize quite naturally to multiphase wavetrains with any finite number of phases (with attendant symmetries and conservation laws). In the case of a simple zero eigenvalue, the results here are almost immediately applicable with some small alterations to accommodate the additional phases. The extra phases also more readily allow for situations where the geometric multiplicity increases and so the coupled KdV emerges as discussed.

There is a dual version of the theory by switching space and time. Then, it is degeneracy of the mapping ***ω***↦**A**(**k**,***ω***) that drives the theory, resulting in a scalar KdV equation with space and time reversed.

## Data accessibility

Although there are no numerical data used in this paper, details of relevant data associated with the publishing and accessibility can be found on the University of Surrey publications repository at http://epubs.surrey.ac.uk.
